# Emerging adults’ use of alcohol and social networking sites during a large street festival: A real-time interview study

**DOI:** 10.1186/s13011-015-0016-3

**Published:** 2015-05-20

**Authors:** Jennifer M. Whitehill, Megan A. Pumper, Megan A. Moreno

**Affiliations:** Department of Health Promotion and Policy, University of Massachusetts Amherst, Amherst, MA USA; Center for Child Health, Behavior and Development, Seattle Children’s Research Institute, Seattle, WA USA; Department of Pediatrics, University of Washington, Seattle, WA USA

**Keywords:** Alcohol, Mifflin Street Block Party, Binge drinking, Drunk driving, Social networking sites, Social media, Facebook, Twitter, Emerging adult

## Abstract

**Background:**

Emerging adults have high rates of heavy episodic drinking (binge drinking) and related risks including alcohol-impaired driving. To understand whether social networking sites (SNSs) used on mobile devices represent a viable platform for real-time interventions, this study measured emerging adults’ use of two popular SNSs (Facebook and Twitter) during the Mifflin Street Block Party. This annual festival is held in Madison, Wisconsin and is known for high alcohol consumption.

**Findings:**

Event attendees ages 18–23 years were recruited by young adult research assistants (>21 years). Participants completed a brief in-person interview assessing drinking intensity, use of SNSs, and use of SNSs to plan transportation. Analyses included t-tests, chi-squared tests, and Fisher’s exact tests. At the event, nearly all of the 200 participants (97 %) consumed alcohol and 18 % met criteria for heavy episodic drinking. Approximately one-third of participants had used Facebook or Twitter on the day of the event. Facebook use (23 %) was more prevalent than Twitter use (18 %), especially among heavy episodic drinkers. Use of either SNS was 41 % among females and 24 % among males (*χ*^2^ = 6.01; df = 1; p = 0.01). Plans to use a SNS to arrange transportation were relatively uncommon (4 %), but this was more frequent among heavy episodic drinkers (11 %) compared to non-heavy episodic drinkers (2 %) (Fisher’s exact p = 0.02).

**Conclusions:**

These results indicate that SNSs are used during alcohol consumption and warrant exploration as a way to facilitate connections to resources like safe ride services.

## Background

Alcohol use is among the leading causes of morbidity and mortality for emerging adults age 18 to 24 years [1]. Approximately 54 % of college students and 50 % of their non-college attending peers report alcohol use in the past 30 days [2]. A particularly risky setting for alcohol consumption and associated negative consequences are alcohol-themed events and parties such as New Year’s Eve, St Patrick’s Day, spring break or Halloween [3, 4]. These events can lead to heavy episodic drinking even among students who typically abstain [3, 5]. Previous work has illustrated associations between heavy alcohol at these events and negative health and behavioral consequences including driving after drinking, and committing acts of theft or vandalism [6].

One specific alcohol-themed event takes place yearly in Madison, Wisconsin. The Mifflin Street Block Party (MSBP), held in May on a major street adjacent to the University of Wisconsin-Madison (UW-Madison) campus, is a day-long event in which 2 blocks of Mifflin Street are blocked-off from vehicular traffic and booths are set up with music and food. In 2011, alcohol was served to those who are over age 21. The street and nearby off-campus student housing become a destination for partying by approximately 10,000–15,000 individuals. A majority of MSBP attendees are students, though other members of the Madison community attend as well. In recent years, the MSBP event has led to arrests for open containers and underage consumption [7, 8], as well as more serious violent offenses [9]. Alcohol-themed events like MSBP occur at many colleges and universities and can present challenges for institutions wishing to keep their students safe from alcohol-related harm [3]. As law enforcement, communities and universities consider novel approaches to appropriately limit or sanction alcohol-themes events, innovative ideas to reduce harm are needed.

Use of online social networking sites (SNS) is widespread among emerging adults and may offer new possibilities for identification of problem drinking and interventions to prevent related consequences during alcohol-themed events. Nearly 75 % of 18–24 year-olds use Facebook [10] and 31 % use Twitter [11], the two most popular SNSs. Young adults with dense online social networks and strong emotional links to their online peers report more alcohol use [12]. Online discussions of substance use have been found to support social norms that are permissive of alcohol use [13]. Research among college students demonstrates that they frequently display references to alcohol consumption on their Facebook profiles [14, 15]. These references are positively associated with clinical measures of problem drinking [16]. A 2014 study in the Wisconsin Medical Journal illustrated that UW-Madison students frequently posted on Facebook about their intention to attend the MSBP and these postings were highly correlated with high levels of alcohol consumption at MSBP [17].

Nearly half of emerging adults own a smartphone that permits mobile use of SNSs [18]. A majority of Facebook users in the United States access the site via a mobile device [19], but previous research has not addressed whether social media is used during episodes of drinking when there are other sources of influence competing in real time for attention (e.g. music, food, friends who are present, etc.) From a public health perspective, the use of SNSs from mobile devices creates the potential to reach individuals where and when they are actively drinking alcohol. Prior qualitative work suggests that some college students thought SNS features could be useful for obtaining help in an emergency or if safe transportation is needed [20], but the extent to which emerging adults use SNSs in real time during alcohol consumption remains unknown. Understanding the characteristics of those who may use SNS in this manner could have implications for targeting efforts to prevent alcohol related harm. Female gender is associated with higher use of SNSs, generally [21], but it is unclear if this holds true in the context of alcohol use. Further, those who engage in heavy episodic drinking are at high risk for alcohol related problems, so it is useful to understand whether that type of drinker is also using SNSs. Thus, the purpose of this exploratory study was to: 1) assess the proportion of emerging adults who use Facebook and/or Twitter during a drinking event, 2) determine whether use of these SNSs varies by gender or drinking intensity, and 3) assess patterns in intent to use SNSs for planning transportation.

## Methods

### Study design

This cross-sectional study utilized participant interviews on-site at a large, alcohol-themed event to capture real-time data.

### Setting

Data were collected during the Mifflin Street Block Party in May 2012. This event had approximately 5,000 attendees, most of whom were college students. This event was an ideal environment in which to study drinking behavior and SNS use in real-time, as it had a significant social media presence with 7,959 “likes” on the event's Facebook page as of November 1, 2012 [22]. Study procedures were approved by the Institutional Review Board at the University of Wisconsin-Madison.

#### Participants

Participants were emerging adults attending the street festival. Inclusion criteria limited participants to those between the ages of 18 and 23 years. Persons who appeared to be heavily intoxicated were not approached.

### Procedure

During the street festival, between 12 pm and 5 pm, 7 research assistants (age > 21) were present at the MSBP in 5 different locations. The research assistants were trained to use a standardized protocol for data collection. The data collectors selected and approached individuals and asked if they were interested in participating in a brief interview about social media and alcohol use. After a script was read, interested individuals provided oral consent. Data collectors read interview questions to the participants and recorded their responses using pen and paper. The interview lasted approximately 5–10 min.

### Data collection

Rather than asking participant age directly, age was assessed as a yes/no response to the question “Are you between the ages of 18 and 23?” in order to avoid disclosures of underage drinking. The interviewer then assessed gender.

To assess alcohol consumption, the interviewer asked the participant if he/she had been drinking any alcohol that day. Participants who indicated they had been drinking were asked: “What time did you begin drinking today?” and “How many drinks have you had so far?” The interviewers recorded the time the interview took place so that the duration of drinking could be calculated.

All participants who indicated that they had been drinking were asked about SNS use. To assess SNS use, drinkers were asked: “Have you used Facebook since you started drinking today?” and “Have you used Twitter since you started drinking today?” Finally, to assess patterns in whether SNS are used for planning safe transportation we asked all participants: “Do you plan to use Facebook or Twitter to arrange transportation home tonight?”

Participants received a small (0.5 L) bottle of water as an incentive for participation.

### Constructed variables

Drinking duration was calculated by subtracting the reported time that drinking began from the time at which the interview was conducted. The rate of drinking was obtained by dividing the number of reported drinks by the duration of drinking. We multiplied the hourly rate by 2 in order to obtain an estimate of heavy episodic drinking that was in line with g criteria from the National Institutes of Alcoholism and Alcohol Abuse (NIAAA), defining heavy episodic drinkers as males who drank at a rate of 5 or more drinks in two hours and females who drank at a rate of more than 4 drinks in 2 h [23] We categorized heavy episodic drinkers as those with a drinking duration of 2 or more hours and a rate greater than or equal to 5 (for males) or 4 (for females) drinks per 2 h.

### Analysis

All analyses were conducted using Stata 12 computer software. Analysis first involved calculating descriptive statistics (means and proportions) for all variables. Chi-square tests were used to assess differences in categorical variables by gender and status as a heavy episodic drinker. Fisher's exact test was used in instances where the expected values were less than 5 observations in at least one cell of the 2 × 2 table. T-tests were used to assess between-group differences for the continuous variables for drinking duration and number of drinks.

## Results

A total of 200 individuals completed the study and 52 % were male. Research assistants approached 214 individuals: 8 people refused to participate (response rate = 96 %) and 6 were ineligible due to being outside the target age range of 18 to 23 years. The majority (97 %) of emerging adults surveyed reported drinking alcohol on the day of the event. On average, participants had been drinking for 5 h (SD = 2.4) and consumed 7.4 drinks (SD = 4.6). Almost one quarter of participants had met NIAAA criteria for heavy episodic drinking (Table 1). Males and females had statistically significant differences in the number of drinks consumed at the event and in the duration of drinking, with males having higher values on both of these measures (Table 2). The proportion of participants who met criteria for heavy episodic drinking was similar for males (19 %) and females (17 %); chi-squared tests indicated this difference was not statistically significant.

**Table 1:** Characteristics of emerging adults age 18-23 years attending the Mifflin Street Block Party (MSBP)

n = 200		n	%
Gender			
	Male	103	52
	Female	97	48
Alcohol use at block party			
	Used alcohol	192	97
	Heavy episodic drinking	43	22
Social networking site use at block party			
	Used Facebook	45	23
	Used Twitter	35	18
	Used either SNS	65	33
Planned to use SNS to arrange transportation from block party		7	4

*SNS* Social networking site. Heavy episodic drinking defined according to NIAAA criteria (>5 drinks in 2 h for males; >4 drinks in 2 h for females)

**Table 2:** Alcohol and SNS use among alcohol-using emerging adults at MSBP, by gender

		Males (n = 99)	Females (n = 93)	*p*-value*
Alcohol use at the block party				
	Mean hours drinking (SD)**	5.5 (2.6)	4.5 (2.0)	0.004
	Mean number of drinks (SD)**	9.6 (5.0)	5.4 (3.0)	<0.001
	% heavy episodic drinkers	19	16	0.696
Social networking site use at the block party				
	% used Facebook	15	31	<0.001
	% used Twitter	14	22	0.142
	% used either SNS	25	42	0.014
	% planned to use SNS for transportation***	3	4	0.715

*SNS* Social networking site, *MSBP* Mifflin Street Block Party

Heavy episodic drinking defined according to NIAAA criteria (>5 drinks in 2 h for males; >4 drinks in 2 h for females)

*p-values reported from chi-squared tests unless otherwise indicated

**p-value from t-tests for difference in means

***p-value from Fisher’s exact test

Overall, 32 % of participants used either Facebook or Twitter since they began drinking that day, with 23 % reporting Facebook use and 18 % reporting Twitter use. Use of both SNSs was higher for females than for males (Table 2). For females, 42 % used either SNS at the event, whereas this was true for only 25 % of males (*χ*^2^ = 6.01; df = 1; p = 0.014).

There were several differences in SNS use based on the intensity of drinking, as shown in Table 3. More heavy episodic drinkers (40 %) used Facebook compared to non-heavy episodic drinkers (19 %) (*χ*^2^ = 7.07; df = 1; p = 0.008). Twitter use was less common among heavy episodic drinkers (9 %) than among non-heavy episodic drinkers (20 %), although this difference was not a statistically significant difference (Fisher’s exact test, p = 0.145).

**Table 3:** Heavy episodic drinking and SNS site use among alcohol-using emerging adults at MSBP

		Heavy episodic drinker (n = 35)	Non-heavy episodic drinker (n = 157)	*p*-value*
Alcohol use at the block party				
	Mean hours drinking (SD)**	3.67 (2.1)	5.35 (2.32)	0.001
	Mean number of drinks (SD)**	10.0 (6.0)	6.9 (4.0)	<0.001
Social networking site use at the block party				
	% used Facebook	40	19	0.008
	% used Twitter***	9	20	0.145
	% used either SNS	46	30	0.086
	% planned to use SNS for transportation***	12	2	0.020

*SNS* Social networking site, *MSBP* Mifflin Street Block Party

Heavy episodic drinking defined according to NIAAA criteria (>5 drinks in 2 h for males; >4 drinks in 2 h for females)

*p-values reported from chi-squared tests unless otherwise indicated

**p-value from t-tests for difference in means

***p-value from Fisher’s exact test

Use of SNS for transportation planning was relatively rare for the sample overall (4 %), but heavy episodic drinkers (12 %) were more likely report intention to use SNS to plan transportation compared to non-heavy episodic drinkers (2 %) (Fisher’s exact test; p = 0.02).

## Discussion and conclusions

To our knowledge, this is the first study to investigate the use of social media during a public event at which alcohol consumption is a primary activity. It builds upon prior knowledge demonstrating that the use of mobile devices and social media are ubiquitous among the emerging adult population [24] by assessing the proportion that use social media while they are actively drinking. The purpose of this study was to assess whether emerging adults use social media during the day of a drinking event, as understanding this could provide a basis for considering future intervention possibilities. The results indicate that 46 % of heavy episodic drinkers used either Facebook or Twitter while drinking on the day of a large alcohol-themed event. Fewer emerging adults used Twitter as compared to Facebook, which is consistent with prior research.

In this study, heavy episodic drinkers, known from previous work to be at elevated risk of alcohol-related motor vehicle crashes, used Facebook during the drinking episode at higher rates than non-heavy episodic drinkers and used Twitter at lower rates. This is consistent with previous studies which showed positive associations between displaying alcohol references on Facebook and self-reported problematic alcohol use [16]. This study extends those findings to illustrate that in some cases, the displayed alcohol references are happening in real-time during a drinking experience.

Use of social media use for transportation planning during this event was relatively low. This may be because many individuals walk to the event and therefore do not spend much time considering transportation. Or, it may reflect the idea that other forms of communication (i.e. phone calls or text messaging) are used to plan transportation rather than social media. This is an area that warrants future exploration. It is possible that if safe ride services were advertised and offered via social media, SNS use to plan transportation would increase.

Heavy episodic drinkers also had a greater tendency to indicate intent to use SNS to plan transportation. Resources to facilitate use of safe transportation, especially delivered through the Facebook environment, may be able to reach a population of high-risk drinkers. One practical translation of this finding could be to encourage event organizers to disseminate messages about a free shuttle bus service or taxi stand locations to individuals who “like” the Facebook page of the alcohol-drinking event. This tactic could allow prevention efforts to be directly and inexpensively targeted to a high-risk population. Mobile users of Facebook, like those in this study, would receive these messages and could potentially even be able to interact with the organizers via Facebook during the event. Future studies could explore the feasibility, acceptability, and outcomes of such an intervention. SNS may also be useful for connecting drinkers to mobile apps such as Uber and Lyft which can be used for arranging safe transportation.

This study has limitations. The potential for selection bias exists because we did not apply standardized random sampling techniques, though we endeavored to sample a variety of event attendees by conducting data collection throughout the duration of the festival and at different locations within the event. The data was self-reported and therefore potentially subject to recall bias. There is also the possibility of social desirability bias, though prior research indicates that young adults disclose heavy episodic drinking behaviors in other in-person assessments [25, 26]. The data collectors were young adults themselves, which may have helped minimize the potential for this bias. It is possible that being under the influence of alcohol could have led participants to over or under-report their alcohol consumption, which is a limitation of any study in which participants have consumed alcohol. Our method for classifying heavy episodic drinkers may have underestimated the number who engaged in this behavior at some point during the day of MSBP. Because the sample was drawn from one event, on one day, the results may not be generalizable outside the context where the study occurred. Efforts to examine whether these results hold true in other settings are encouraged.

In conclusion, nearly one-third of emerging adults used SNSs while drinking on the day of MSBP, an alcohol-themed event; those who drank heavily were more likely to use Facebook compared to those who did not drink heavily. This finding should motivate universities and event organizers to incorporate social media, especially Facebook, into their plans for advertising harm reduction strategies and offering safe ride services when alcohol-themed events take place. Additional research will be needed to evaluate the reach and impact of such efforts. Nonetheless, this study is an important first step towards understanding the potential for targeted, real-time interventions that use social media to influence behavior related to risky alcohol consumption and alcohol impaired driving.

## Abbreviations

SNS: Social networking site
MSBP: Mifflin Street Block Party
SMS: Short message service
L: Liter
NIAAA: National Institutes of Alcoholism and Alcohol Abuse
